# Efficacy of a Modified Treat-and-Extend Aflibercept Regimen for Macular Oedema in Eyes with Central Retinal Vein Occlusion: 2-Year Prospective Study

**DOI:** 10.3390/jcm12155089

**Published:** 2023-08-02

**Authors:** Yusuke Arai, Hidenori Takahashi, Satoru Inoda, Shinichi Sakamoto, Xue Tan, Hidetoshi Kawashima, Yasuo Yanagi

**Affiliations:** 1 Department of Ophthalmology, Jichi Medical University, 3311-1 Yakushiji, Shimotsuke City 329-0498, Tochigi, Japan; r1003ya@jichi.ac.jp (Y.A.); r1208is@jichi.ac.jp (S.I.); r1136ss@jichi.ac.jp (S.S.); hidemeak@khaki.plala.or.jp (H.K.); 2 Japan Community Health Care Organization Tokyo Shinjuku Medical Center, 5-1 Tsukudocho, Shinjuku-ku, Tokyo 162-8543, Japan; tanxue1201@hotmail.com; 3 Department of Ophthalmology and Micro-Technology, Yokohama City University, 4-57 Urafunecho, Minami-ku, Yokohama City 232-0023, Kanagawa, Japan; yanagi.yasuo@icloud.com

**Keywords:** central retinal vein occlusion, anti-vascular endothelial growth factor, modified treat-and-extend regimen, personalized therapy, prospective multicenter intervention study

## Abstract

This prospective, multicentre, interventional study evaluated the efficacy of a modified treat-and-extend (mTAE) aflibercept regimen as personalized therapy for macular oedema (MO) due to central retinal vein occlusion (CRVO). Fifty eyes were studied from 50 patients who were enrolled between November 2016 and July 2019. All patients received intravitreal aflibercept (IVA) injections on an mTAE regimen for 24 months. Primary outcome measures were best-corrected visual acuity (BCVA) and central subfield thickness (CST) at 12 months. Secondary endpoints were BCVA and CST at 24 months. Mean (standard deviation) baseline BCVA (logMAR) and CST were 0.50 (0.51) and 557 (240) µm, respectively. BCVA and CST showed significant improvements at month 12 (0.19 (0.38) and 275 (98) µm, respectively; both *p* < 0.0001, paired *t*-test). BCVA and CST also showed significant improvements at 24 months (0.26 (0.50) and 255 (91) µm, respectively, *p* = 0.0004 and *p* < 0.0001, paired *t*-test). The mean numbers of IVA injections and clinic visits over the 24-month study period were 6.2 (3.0) and 10.3 (1.0), respectively. The mTAE regimen of IVA injections for MO due to CRVO was effective in improving BCVA and decreasing CST at 24 months. The mTAE regimen might be an effective personalized therapy for CRVO.

## 1. Introduction

Central retinal vein occlusion (CRVO) is among the most common retinal vascular diseases that cause significant vision loss. After the development of CRVO, vision loss is usually due to macular oedema (MO), macular ischaemia, or in more advanced stages, the development of foveal pigmentation, an epiretinal membrane, and neovascular glaucoma [1]. Anti-vascular endothelial growth factor (VEGF) therapy is currently the standard of care for MO secondary to CRVO [2]. The efficacy of anti-VEGF in CRVO has been shown in numerous studies [3,4,5]. COPERNICUS and GALILEO were the first large-scale studies and initially used 6-monthly injections of aflibercept [4,5], which achieved excellent structural and functional improvements during the loading period and monthly follow-up period. However, the results of the HORIZON study suggest that the frequency of follow-up and intravitreal injections in patients with retinal vein occlusion should be individualized for effective treatment and that patients with CRVO in particular may require follow-ups more frequently than every 3 months [6].

Campochiaro et al. [7] found no difference between patients treated according to a pro re nata (PRN) regimen and those treated monthly. However, the PRN regimen remains based on monthly clinical examinations. The treat-and-extend (TAE) regimen is an individualized treatment regimen that aims to reduce the burden due to frequent treatments, specifically the number of visits [8,9]. This regimen is now commonly used to treat age-related macular degeneration (AMD).

There are some reports indicating that the TAE regimen is effective for MO due to CRVO [10,11]. However, some patients with cystoid macular oedema have been found not to relapse after a single injection [7], which suggests that treatment with the TAE regimen following the first injection may be overtreatment as the TAE regimen requires a second injection 1 month after the first injection. Accordingly, we considered it necessary to develop an initial treatment strategy that can reduce the number of initial doses administered in the TAE regimen for MO due to CRVO. In the present study, the second injection was given according to the results of monitoring for recurrence of MO at 4-week intervals. Thereafter, the injection interval was adjusted as needed. In an earlier study, we prospectively evaluated the efficacy and safety of this modified TAE (mTAE) regimen in patients with BRVO [12,13]. This mTAE regimen is designed to minimize the number of hospital visits and injections. In the present report, we present the 24-month results for intravitreal aflibercept (IVA) injections administered according to this mTAE regimen in patients with MO due to CRVO.

## 2. Materials and Methods

### 2.1. Patients and Approval

The participants in this prospective, multicentre, interventional study were consecutive patients with treatment-naïve MO due to CRVO. Enrolment started in October 2016 and the last patient completed the 1-year study in July 2020. The study was carried out at 4 institutions in Japan (Jichi Medical University Hospital, Japan Community Health Care Organization Tokyo Shinjuku Medical Center, Takahashi Eye Clinic, and Aoki Eye Clinic). The study protocol was approved by the institutional review board of Jichi Medical University (B18-004) and adhered to the tenets of the Declaration of Helsinki. Informed consent was obtained from all patients who participated in the study. The study was registered in the University Hospital Medical Information Network Clinical Trials registry prior to the enrolment of the first patient (UMIN000024588; 27 October 2016). No clear sex differences have been reported in RVO [14]. In the present analysis population, the difference in sex ratio was evaluated using the chi-square test, and the prognostic impact on visual acuity and CST was evaluated by multivariate analysis including sex.

### 2.2. Methods and Ophthalmic Examination

Inclusion criteria were age 20–89 years and a diagnosis of MO that was central and secondary to treatment-naïve CRVO within the 12 months before screening. The following exclusion criteria were applied: (1) history of prior anti-VEGF treatment or intravitreal or sub-Tenon corticosteroid therapy; (2) history of vitreoretinal surgery; (3) history of fundus photocoagulation treatment; (4) history of intraocular surgery (without YAG capsulotomy) within 3 months of enrolment; (5) other disease that could cause decreased visual acuity (except for mild or moderate cataracts); and (6) history of thromboembolic events within 3 months of enrolment. Eyes with hemi-CRVO were included.

A complete ophthalmic examination was performed for all patients and included assessment of BCVA with refraction using the 5M Landolt C VA chart, measurement of intraocular pressure, and evaluation of central subfield thickness (CST) using spectral-domain optical coherence tomography (OCT), swept-source OCT, indirect ophthalmoscopy, and fundus photography at all visits. Table S1 shows the OCT devices and fundus cameras used in this study. No eligibility criteria restricted entry based on BCVA and CST. Fluorescein angiography (FA) was carried out prior to the first injection except in patients with a fluorescein allergy and those for whom the investigator deemed FA to be high risk. MO was diagnosed based on OCT and fundus findings. FA was repeated at months 12 and 24.

### 2.3. mTAE Regimen

Aflibercept treatment was performed in accordance with the mTAE regimen, the details of which have been described previously [12,13]. No initial dosing was administered. All patients received the first injection and then attended follow-up visits every 4 weeks. If there was any exudative lesion in the macula (MO and/or serous retinal detachment in any of the serial macular OCT scans), the second injection was given and then the TAE process was started. At that time, there were no CST criteria. The second injection criterion was any exudative lesion in any of the serial macular OCT scans. After the second injection, if there was no exudative lesion in any of the OCT scans, the eye was defined as having a dry macula, IVA was given, and the period to the next treatment was extended by 2 weeks at a time (no maximum interval specified) [12,13]. After the first injection, if a dry macula was maintained (defined as the absence of exudative lesions), follow-up visits were continued every 4 weeks until week 16, after which the interval between visits could be extended to 3 months. When an exudative lesion in the macula (MO and/or serous retinal detachment in any of the serial macular OCT scans) was found, a second injection was administered and then the TAE process was started. Up to week 16, this process was as follows: If the macula was dry (no maximum specified), an injection was given and the follow-up interval was extended by 2 weeks. If exudative lesions had worsened compared with the previous visit, an injection was administered and the follow-up interval was shortened by 2 weeks. If an exudative lesion had not worsened since the last visit, an injection was administered and the interval remains unchanged. If the first recurrence occurred after week 16, a TAE regimen was started with 3-month intervals. Examples of the mTAE regimen in different patients are shown in Figures S1–S3. Examples of PRN and TAE regimens compared with the mTAE regimen are shown in Figure S4a,b.

### 2.4. Outcome Measures

The primary outcome measures were the mean changes in BCVA and CST at month 12. Secondary outcome measures were BCVA and CST at month 24. In addition, we also investigated the number of IVA injections and the number of clinic visits during the 2-year study period. In addition, we examined the time to the first recurrence from the initial IVA injection. Changes in BCVA and CST and the number of clinic visits and injections were compared between eyes with CRVO and those with hemi-CRVO.

### 2.5. Statistical Analysis

Statistical analysis was carried out using JMP Pro software version 15.1.0 (SAS Institute, Cary, NC, USA). Decimal BCVA was converted to the logarithm of the minimum angle of resolution (logMAR). BCVA and CST were compared with their respective values at the time of the first injection using two-tailed paired *t*-tests with Bonferroni correction. The efficacy endpoints were analysed in a full analysis set, which included all patients who received any study treatment and had one baseline and at least one post-baseline assessment. Between months 4 and 23, missing data were imputed by the last observation carried forward method. Moreover, we used multivariate analysis to determine whether baseline BCVA, CST, and number of injections were associated with improvements in BCVA and CST at 24 months. A *p*-value of less than 0.05 was considered statistically significant.

## 3. Results

### 3.1. Baseline Characteristics

**Figure 1:**
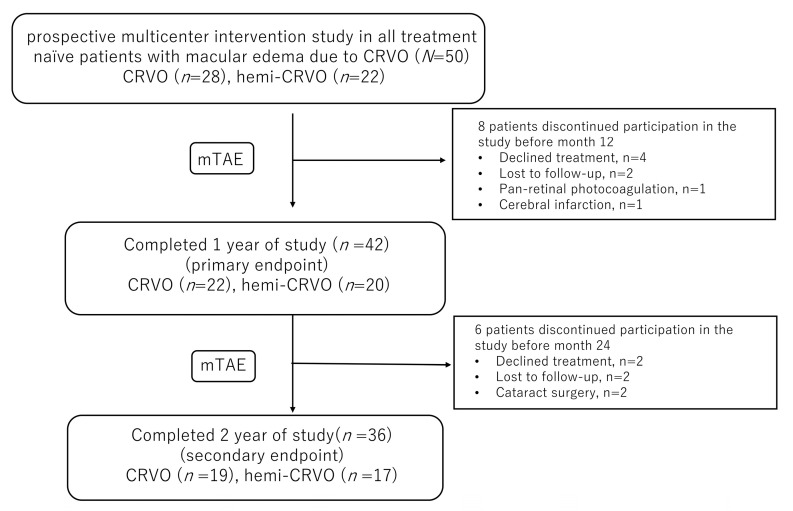
The study design and patient disposition.

Fifty eyes of 50 patients (24 men, 26 women) were enrolled in the study (Table 1). Hemi-CRVO was present in 22 eyes. There were 14 dropouts (six declined treatment, four were lost to follow-up, two underwent cataract surgery, one underwent pan-retinal photocoagulation, and one had a cerebral infarction). Nine of the 14 patients who dropped out had CRVO. Thus, 19 of the remaining 36 eyes had CRVO (Figure 1). The mean patient age was 70 (standard deviation, 12) years (range, 34–87) at the start of treatment. Mean baseline BCVA (logMAR) was 0.50 (0.51) and the mean baseline CST was 557 (240) µm. FA revealed that ischaemic CRVO was present in four eyes before the first injection.

### 3.2. VA Outcomes and CST

**Figure 2:**
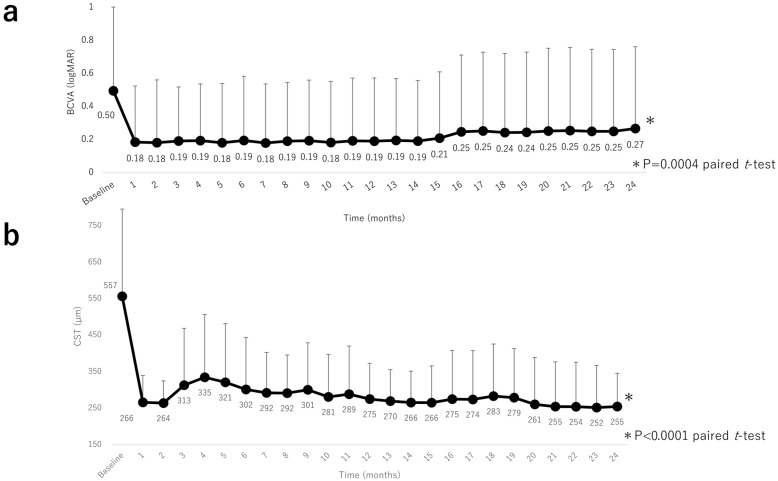
Visual acuity outcomes and central subfield thickness over 24 months. (**a**) Changes in mean BCVA (logMAR) during the 24-month study period. There was a significant improvement in mean BCVA by month 1 after intravitreal aflibercept injection that continued through to month 24. (**b**) Changes in mean CST during the 24-month study period. There was a significant improvement in mean CST by month 1 after intravitreal aflibercept injection that was sustained through to month 24. BCVA, best-corrected visual acuity; logMAR, logarithm of the minimum angle of resolution, CST, central subfield thickness.

Mean BCVA improved significantly from 0.50 (0.51) at baseline to 0.18 (0.34) at 1 month, 0.19 (0.38) at 12 months, and 0.26 (0.50) at 24 months (all *p* < 0.0001, paired *t*-test; Figure 2a). We examined the percentage of eyes with improvement of ≥15 letters in the ETDRS score. Improvement was seen in 18 eyes (50%). CST decreased significantly from 557 (240) µm at baseline to 266 (74) µm at 1 month, 275 (98) µm at 12 months, and 255 (91) µm at 24 months; all *p* < 0.0001, paired *t*-test; Figure 2b). Multivariate analysis revealed that an improvement in visual acuity at 24 months was significantly correlated with a lower baseline BCVA (*p* = 0.0006). There was no correlation between baseline CST and the number of injections (*p* = 0.80 and *p* = 0.99, respectively). In addition, an improvement in CST at 24 months was significantly correlated with a greater baseline CST (*p* < 0.0001). There was no correlation between baseline BCVA and the number of injections (*p* = 0.24 and *p* = 0.22, respectively).

### 3.3. Mean Number of IVA Injections and Clinic Visits

**Figure 3:**
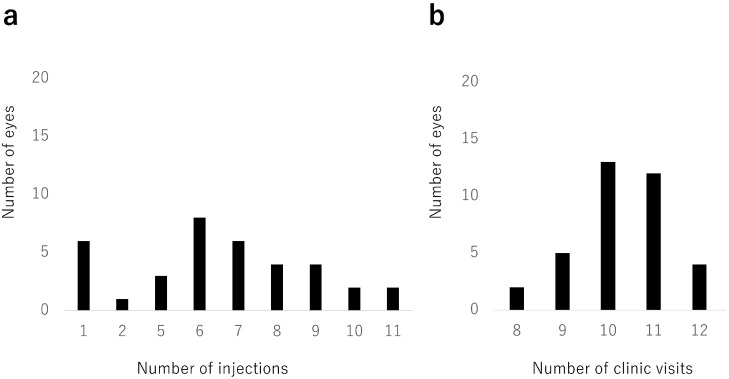
Number of injections and clinic visits. (**a**) Distribution of numbers of intravitreal aflibercept injections. Six eyes (17%) received only 1 injection. The mean number of intravitreal aflibercept injections was 6.2 (standard deviation, 3.0) during the 24-month study period. (**b**) Distribution of numbers of clinic visits. The mean number of clinic visits was 10.3 (standard deviation, 1.0) during the 24-month study period.

The mean number of IVA injections during the 2-year study period was 6.2 (3.0) (Figure 3a). Six eyes received only one injection; only one of these eyes had hemi-CRVO. The mean number of clinic visits was 10.3 (1.0) over 2 years (Figure 3b).

### 3.4. Time to First Recurrence after the First Injection

**Figure 4:**
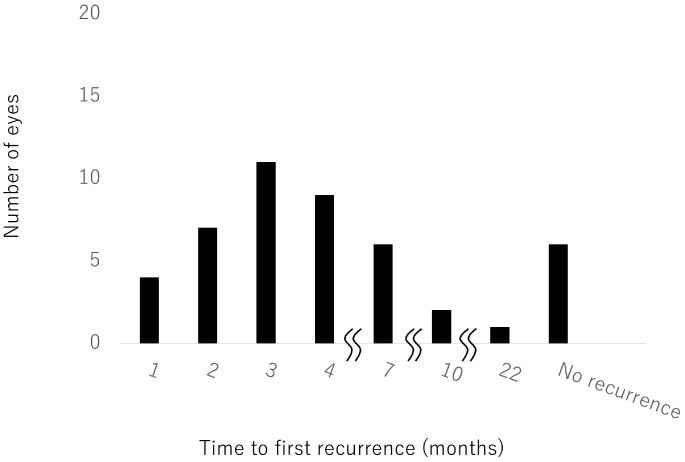
Time to first recurrence after the first injection.

Time to first recurrence after the first injection could be examined in 46 eyes (Figure 4). Recurrence was most common at 3 months (11 eyes, 24%). Nine eyes had a first recurrence after more than 6 months. There was no recurrence in six eyes, only one of which had hemi-CRVO. When we compared baseline characteristics between patients with ≥7 injections and patients with ≤6 injections, we found that baseline CST was significantly smaller in the group with ≤6 injections. There were no other significant differences between these groups.

### 3.5. Comparison between CRVO and Hemi-CRVO

The characteristics of eyes with CRVO and those with hemi-CRVO are compared in Table 2.

**Figure 5:**
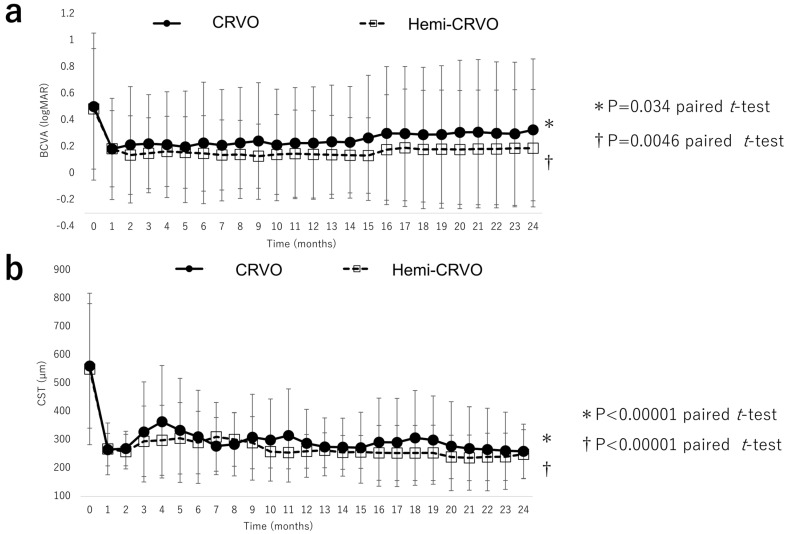
Visual acuity outcomes and central subfield thickness of CRVO and hemi–CRVO. (**a**) Changes in mean BCVA (logMAR) in eyes with CRVO and eyes with hemi–CRVO during the 24-month study period. There were significant improvements in mean BCVA in patients with CRVO and those with hemi–CRVO at month 24. There was no significant between-group difference. (**b**) Changes in central subfield thickness in eyes with CRVO and eyes with hemi–CRVO during the 24-month study period. There were significant improvements in mean central subfield thickness in patients with CRVO and those with hemi–CRVO at month 24. There was no significant between-group difference. BCVA, best-corrected visual acuity; CRVO, central retinal vein occlusion; logMAR, logarithm of the minimum angle of resolution.

Figure 5 shows the improvements in BCVA and CST in patients with CRVO and those with hemi-CRVO. The mean BCVA in eyes with CRVO and those with hemi-CRVO improved significantly from 0.50 (0.55) and 0.49 (0.45), respectively, at 12 months (0.23 [0.42] and 0.14 [0.33]) and 24 months (0.33 [0.53], and 0.19 [0.44]; both *p* < 0.0001, paired *t*-test). Mean CST in eyes with CRVO and eyes with hemi-CRVO decreased significantly from 561 (220) µm and 550 (268) µm, respectively, at baseline to 288 (120) µm and 259 (59) µm at 12 months and to 259 (96) µm and 249 (87) µm at 24 months; both *p* < 0.0001, paired *t*-test). BCVA and CST at 24 months did not significantly differ between eyes with CRVO and those with hemi-CRVO (*p* = 0.33 and *p* = 0.69, respectively; non-paired *t*-test).

There was no significant difference in the mean number of IVA injections administered to eyes with CRVO and those with hemi-CRVO (5.9 (3.5) and 6.5 (2.3), respectively; *p* = 0.77, non-paired *t*-test). There was also no significant difference in the mean number of visits between patients with CRVO and those with hemi-CRVO (10.5 (1.1) and 10.1 (1.0), respectively; *p* = 0.26, non-paired *t*-test).

### 3.6. Ischaemic CRVO and Neovascular Glaucoma

There were no cases of neovascular glaucoma during the study period. FA was performed in 25 eyes at month 24. Ischaemic CRVO was observed in eight eyes, including three in which it was present before treatment. The mean BCVA at month 24 was 0.20 (0.32) in eyes with ischaemic CRVO and 0.02 (0.17) in those with non-ischaemic CRVO. The mean CST was 243 (64) µm in eyes with ischaemic CRVO and 271 (57) µm in those with non-ischaemic CRVO. At month 24, BCVA was significantly better in non-ischaemic CRVO than in ischaemic CRVO (*p* = 0.03; non-paired *t*-test), but there was no significant difference in CST between these groups (*p* = 0.18; non-paired *t*-test). Moreover, there was also no significant difference in the mean number of injections between patients with ischaemic CRVO and those with non-ischaemic CRVO (6.9 [2.3] and 5.6 [3.0], respectively; *p* = 0.58, non-paired *t*-test).

### 3.7. Safety Outcomes at 24 Months

The IVA injections were not associated with any serious ocular complications. One serious systemic adverse event occurred in a patient who developed a renal infarction 2 months after IVA and a cerebral infarction 3 months after IVA. The relationship with IVA was unknown.

## 4. Discussion

The results of this study demonstrated the short-term and long-term efficacy of the mTAE regimen of IVA injections for treating MO due to CRVO in terms of improvement of visual acuity and reduction of CST. The mTAE regimen used in this prospective study is the first to combine the best of the PRN and TAE regimens for CRVO. The BCVA and CST values were consistent with those of the pivotal studies, namely, COPERNICUS and GALILEO [4,5]. Moreover, the ETDRS score improved by ≥15 letters in 50% of patients in this study, similar to the result in the COPERNICUS study [4]. In those studies, the gains in visual acuity achieved by six-monthly aflibercept injections were largely maintained for up to 1 year using PRN injections [5,15]. However, the results of COPERNICUS suggested the need for more frequent monitoring for the prevention of disease because of decreases in the visual and anatomic gains seen during the second year with PRN dosing and during quarterly evaluations [4]. In our present study, improvements in both BCVA and CST were seen at month 1 and maintained thereafter. Moreover, even with the smaller number of injections, the functional results in our study were comparable to the large-scale multicentre prospective results. We believe that this was for the following reasons: (1) the mTAE regimen was used to provide initial treatment with fewer injections; (2) proactive treatment prevented the recurrence of oedema in the TAE phase; and (3) pre-treatment CST was less in our study than in large clinical studies [4,5], so our study might have had fewer severe cases. The CRYSTAL study, on the other hand, investigated a PRN regimen using ranibizumab and found improvements in both BCVA and CST that were maintained for 2 years [16]. Monthly monitoring was very useful. However, the RETAIN study showed that half of the eyes still required anti-VEGF injections at regular intervals during long-term follow-up [17]. Continuing monthly clinic visits may increase the burden on patients. In our study, the time to first recurrence and the interval between IVA injections during the TAE period varied from patient to patient. The reason for the difference is unknown but may be related to differences in the degree of obstruction, VEGF concentration, and inflammatory cytokine concentrations. Therefore, an individualized regimen is likely needed for MO due to CRVO. In the present study, improvements in BCVA and CST were associated with poor baseline BCVA and thicker CST, similar to previously reported findings [18] Moreover, baseline CST was significantly smaller in patients with ≤6 injections. Patients with smaller baseline CST may require fewer injections.

A TAE regimen aims to achieve visual outcomes similar to those of the more intensive regimens but with fewer injections and clinic visits, as has been demonstrated in the management of neovascular AMD [19,20]. In our study, there was no recurrence in six eyes. Therefore, we believe that choosing a strict TAE regimen at the outset and three monthly loading doses may lead to overtreatment in some patients, and this can be avoided by the mTAE regimen. However, as shown in this study, some cases do not relapse for up to 16 weeks after a first injection. The mTAE regimen individualizes treatment intervals and can potentially prevent undertreatment. Shimura et al. have reported an individualized regimen for MO due to CRVO [21]. In their protocol, the response of MO to pharmacological treatment was defined as the time between the initial injection and recurrence of oedema (called the period of efficacy [POE]), which was determined by monitoring patients every 2 weeks. After recurrence, the treatment interval for an individual patient was defined as 1 week less than the POE for that patient. Their report showed that both BCVA and CMT were significantly better at 24 months than at baseline. The 42 eyes with recurrence required a mean of 8.3 aflibercept injections and 14.4 visits over the 24-month study period. During our 2-year study, a mean of 6.2 IVA injections were administered at 10.3 (1.0) clinic visits. Therefore, our mTAE regimen may be more efficient and less burdensome for patients than the POE regimen.

In our previous study of the mTAE regimen for MO due to BRVO, we found that there was no further recurrence if the first recurrence did not occur until 4 months after the first injection [13]. However, in our present study, the first recurrence did not occur until more than 6 months after the first IVA injection in nine eyes. Therefore, careful monitoring is needed because patients with CRVO can develop recurrence long after the first injection. It has been reported that patients with CRVO have very high levels of intravitreal VEGF [22,23,24], and the intravitreal VEGF concentration differs between BRVO and CRVO [23]. The size of the ischaemic area correlates with VEGF concentration in BRVO [25]. This difference may explain why MO in CRVO can occur more than 4 months after the first injection.

In this study, no differences were observed between CRVO and hemi-CRVO. Koss et al. reported that cytokine values were more similar between eyes with hemi-CRVO and those with CRVO than between eyes with hemi-CRVO and those with BRVO [21]. In our study, there was no significant difference in BCVA, CST, the mean number of IVA injections, or the number of clinic visits between CRVO and hemi-CRVO. CRVO and hemi-CRVO may be considered not only in terms of cytokine values but also in terms of clinical characteristics. Ischaemic CRVO was observed in eight eyes at month 24, but none of these cases progressed to neovascular glaucoma. There may be less concern about neovascular glaucoma in patients receiving IVA injections. However, when the TAE period ends, it is important to determine whether CRVO is ischaemic or non-ischaemic. In this study, BCVA was significantly better in non-ischaemic CRVO than in ischaemic CRVO, but there was no difference in CST and number of injections. The presence of macular ischaemia may have a significant impact on visual acuity. However, the results suggest that mTAE regimens can improve CST with fewer injections for both ischaemic and non-ischaemic CRVO.

This study has some limitations. First, it had a single-arm design. Second, the sample size was relatively small. Third, only Japanese patients were included. To confirm its utility, the mTAE regimen should be examined in larger prospective studies with longer follow-up durations. Moreover, the efficacy of the mTAE regimen should be compared with that of other administration methods in a prospective study.

## 5. Conclusions

In this prospective study, an mTAE regimen of IVA injections for MO due to CRVO improved BCVA and reduced CST over a period of 2 years. The mTAE regimen is effective for improving BCVA and structural outcomes as well as for reducing the number of injections required and the number of clinic visits. A novel aspect of the present mTAE regimen is that it includes a monitoring phase in which the treatment interval can be personalized based on disease activity. We believe that the present regimen may reduce the burden on patients by minimizing the number of injections and reducing the number of hospital visits while maintaining the improvement in BCVA.

## Figures and Tables

**Table 1:** Characteristics of enrolled patients.

Cases	50
Age (years; mean (SD, range))	70 (12, 34–93)
Sex (male/female)	32/18
BCVA (logMAR; mean (SD))	0.50 (0.51)
CST (µm; mean (SD, range, median))	557 (240, 217–1302, 522)
Lens (phakic/intraocular lens)	42/8

BCVA, best-corrected visual acuity; CST, central subfield thickness; SD, standard deviation.

**Table 2:** Comparison of CRVO and hemi-CRVO.

	CRVO (*n* = 28)	Hemi-CRVO (*n* = 22)
Age (years; mean (SD, range))	68.9 (13.8, 34–89)	70.6 (10.2, 54–93)
Sex (male)	14	18
BCVA (logMAR; mean (SD))	0.50 (0.55)	0.49 (0.45)
CRT (µm; mean (SD, range, median))	561 (220, 289–1190, 242)	550 (268, 217–1302, 259)
Lens (phakic/intraocular lens)	23/5	19/3

BCVA, best-corrected visual acuity; CRVO, central retinal vein occlusion; CST, central subfield thickness; SD, standard deviation.

## Data Availability

All data set files are available from the Figshare database (https://doi.org/10.6084/m9.figshare.23306102, accessed on 7 June 2023).

## Supplementary Materials

The following supporting information can be downloaded at: https://www.mdpi.com/article/10.3390/jcm12155089/s1, Table S1: Fundus cameras and optical coherence tomography systems at each institution; Figure S1: Criteria for starting retreatment at month 3; Figure S2: Criteria for starting the treat-and-extend regimen at month 4; Figure S3: Criteria for starting the treat-and-extend regimen for recurrence after the end of monthly examinations; Figure S4: (a) Pro re nata regimen. (b) Treat-and-extend regimen (2-week intervals).
