# Supplementary material for: Efficacy of a Modified Treat-and-Extend Aflibercept Regimen for Macular Oedema in Eyes with Central Retinal Vein Occlusion: 2-Year Prospective Study
Source: J Clin Med. 2023 Aug 2;12(15):5089. doi: 10.3390/jcm12155089 (PMC10419814; doi:10.3390/jcm12155089)
Supplement: Supplementary file 1 [file jcm-12-05089-s001.zip › jcm-2465939-supplementary.pdf]

Journal of
Clinical Medicine

MDPI

Supplementary Materials for

Efficacy of a Modified Treat-and-Extend Aflibercept Regimen for Macular Oedema in Eyes with Central Retinal Vein Occlusion: 2-Year Prospective Study

Yusuke Arai [1], Hidenori Takahashi [1,*], Satoru Inoda [1], Shinichi Sakamoto [1], Xue Tan [2], Hidetoshi Kawashima [1] and Yasuo Yanagi [3]

[1] Department of Ophthalmology, Jichi Medical University, 3311-1 Yakushiji, Shimotsuke City 329-0498, Tochigi, Japan; r1003ya@jichi.ac.jp (Y.A.); r1208is@jichi.ac.jp (S.I.); r1136ss@jichi.ac.jp (S.S.); hidemeak@khaki.plala.or.jp (H.K.)
[2] Japan Community Health Care Organization Tokyo Shinjuku Medical Center, 5-1 Tsukudocho, Shinjuku-ku, Tokyo 162-8543, Japan; tanxue1201@hotmail.com
[3] Department of Ophthalmology and Micro-Technology, Yokohama City University, 4-57 Urafunecho, Minami-ku, Yokohama City 232-0023, Kanagawa, Japan; yanagi.yasuo@icloud.com
* Correspondence: takahah-tky@umin.ac.jp; Tel.: +81-285-58-7382

Contents

Table S1. Fundus cameras and optical coherence tomography systems at each institution.

Figure S1. Criteria for starting retreatment at month 3.

Figure S2. Criteria for starting the treat-and-extend regimen at month 4.

Figure S3. Criteria for starting the treat-and-extend regimen for recurrence after the end of monthly examinations.

Figure S4. (a) *Pro re nata* regimen. (b) Treat-and-extend regimen (2-week intervals).

Table S1. Fundus cameras and optical coherence tomography systems at each institution.

Institution	Camera	OCT
A	VX-10	RS-3000, DRI OCT Triton
B	TRC-50DX Type IA	Cirrus HD-OCT Model 4000
C	VX-10	Cirrus HD-OCT Model 4000
D	Optos200Tx	RS-3000

Institution A: Jichi Medical University Hospital; B: Japan Community Health Care Organization Tokyo Shinjuku Medical Center; C: Takahashi Eye Clinic; D: Saito Eye Clinic, F: Aoki Eye Clinic
VX10: Kowa Co Ltd., Tokyo, Japan. TRC-50DX Type1A, DRI OCT Triton: Topcon Medical Systems, Tokyo, Japan. Optos200Tx: Optos plc, Dunfermline, UK. RS-3000: NIDEK CO., LTD, Tokyo, Japan. HD-OCT Model 4000: Carl Zeiss Meditec, Jena, Germany

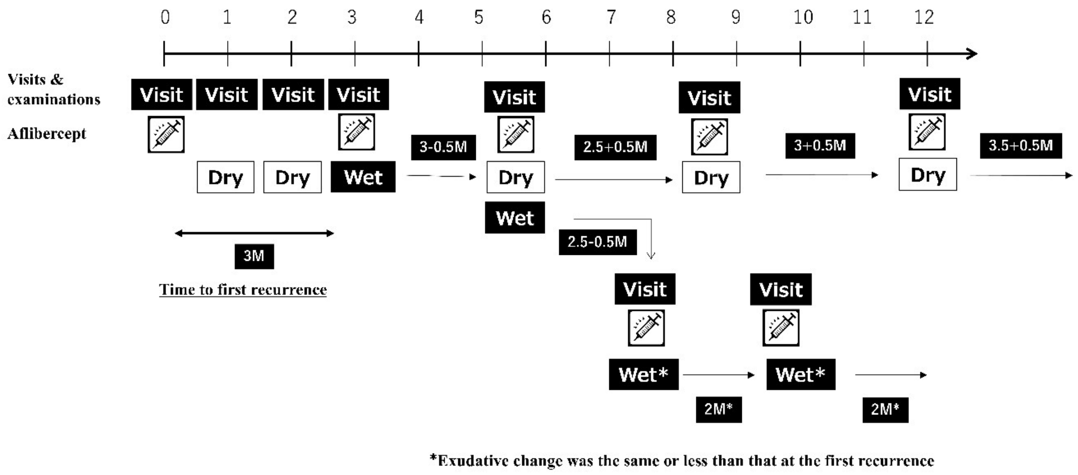

Figure S1. Criteria for initial treatment at month 3.

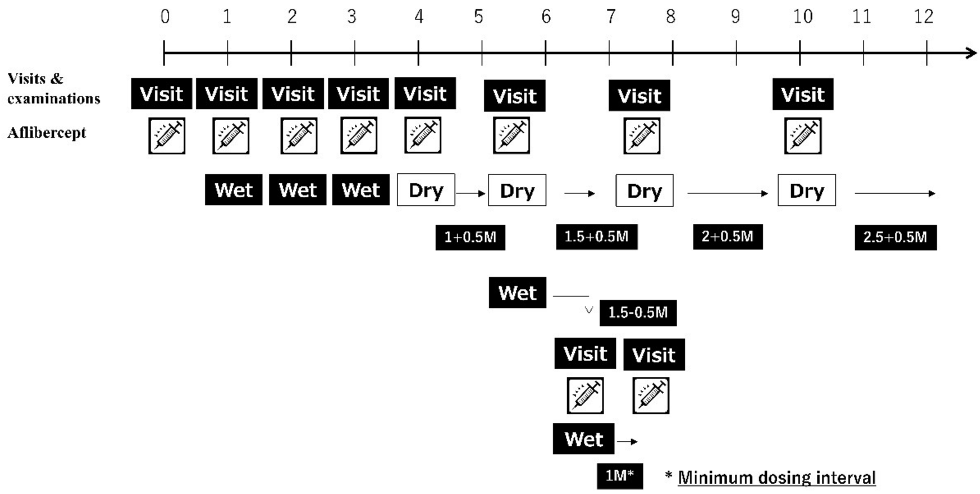

Figure S2. Criteria for initiating the treat-and-extend regimen at month 4.

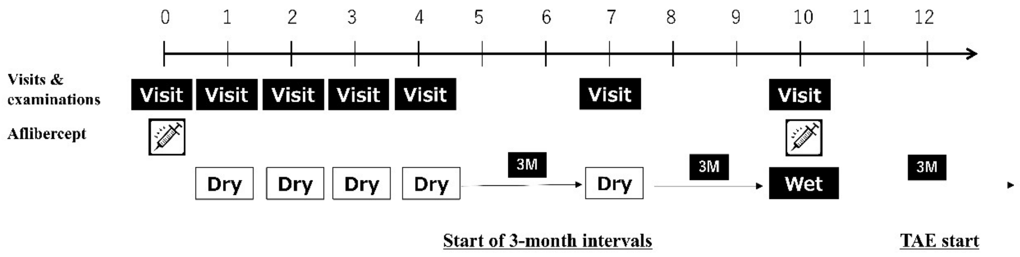

Figure S3. Criteria for starting the treat-and-extend (TAE) regimen for recurrence after the end of monthly examinations.

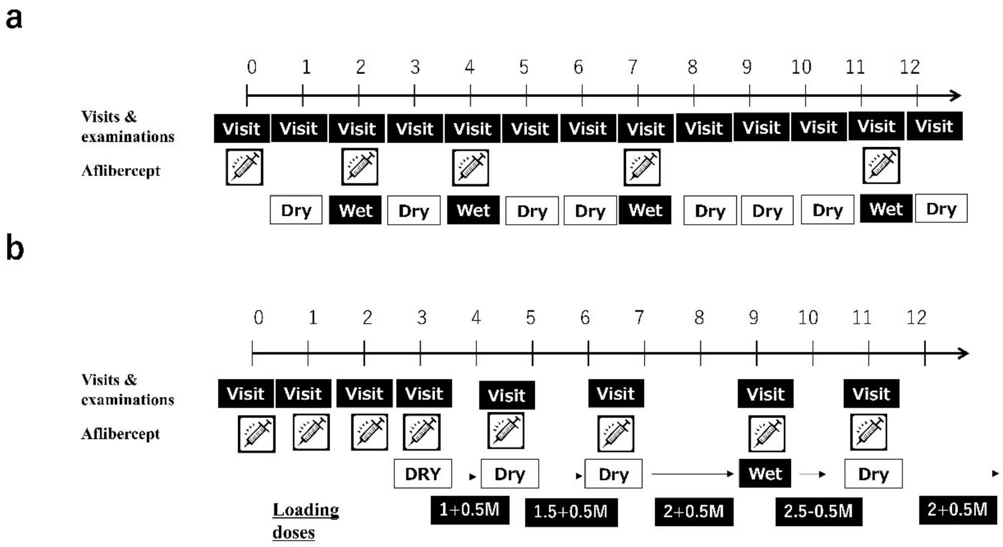

Figure S4. (a) *Pro re nata* regimen. (b) Treat-and-extend regimen (2-week intervals).
